# Low Power Operation of Temperature-Modulated Metal Oxide Semiconductor Gas Sensors

**DOI:** 10.3390/s18020339

**Published:** 2018-01-25

**Authors:** Javier Burgués, Santiago Marco

**Affiliations:** 1 Department of Electronic and Biomedical Engineering, Universitat de Barcelona, Marti i Franqués 1, 08028 Barcelona, Spain; smarco@ibecbarcelona.eu; 2 Signal and Information Processing for Sensing Systems, Institute for Bioengineering of Catalonia (IBEC), The Barcelona Institute of Science and Technology, Baldiri Reixac 10-12, 08028 Barcelona, Spain

**Keywords:** smartphone, metal-oxide semiconductor, gas sensor, low power, temperature-modulation, interferences

## Abstract

Mobile applications based on gas sensing present new opportunities for low-cost air quality monitoring, safety, and healthcare. Metal oxide semiconductor (MOX) gas sensors represent the most prominent technology for integration into portable devices, such as smartphones and wearables. Traditionally, MOX sensors have been continuously powered to increase the stability of the sensing layer. However, continuous power is not feasible in many battery-operated applications due to power consumption limitations or the intended intermittent device operation. This work benchmarks two low-power, duty-cycling, and on-demand modes against the continuous power one. The duty-cycling mode periodically turns the sensors on and off and represents a trade-off between power consumption and stability. On-demand operation achieves the lowest power consumption by powering the sensors only while taking a measurement. Twelve thermally modulated SB-500-12 (FIS Inc. Jacksonville, FL, USA) sensors were exposed to low concentrations of carbon monoxide (0–9 ppm) with environmental conditions, such as ambient humidity (15–75% relative humidity) and temperature (21–27 °C), varying within the indicated ranges. Partial Least Squares (PLS) models were built using calibration data, and the prediction error in external validation samples was evaluated during the two weeks following calibration. We found that on-demand operation produced a deformation of the sensor conductance patterns, which led to an increase in the prediction error by almost a factor of 5 as compared to continuous operation (2.2 versus 0.45 ppm). Applying a 10% duty-cycling operation of 10-min periods reduced this prediction error to a factor of 2 (0.9 versus 0.45 ppm). The proposed duty-cycling powering scheme saved up to 90% energy as compared to the continuous operating mode. This low-power mode may be advantageous for applications that do not require continuous and periodic measurements, and which can tolerate slightly higher prediction errors.

## 1. Introduction

Market forecasts [1] indicate that the number of portable air quality devices will increase with respect to fixed air quality stations over the next few years. The high market penetration of smartphones [2] and wearables has opened a market for mobile applications based on gas sensing. Adding a chemical analysis capability to smartphones targets the in situ detection of Volatile Organic Compounds (VOCs). Very low levels of VOCs have been found to contribute to the “sick building syndrome”, which degrades workers’ health and decreases productivity [3]. At higher concentrations, both short- and long-term exposure to VOCs are known to create health risks [4]. The World Health Organization (WHO) has identified nine harmful compounds, including carbon monoxide (CO), benzene, nitrogen dioxide (NO_2_), formaldehyde, and naphthalene, which might be present in indoor air [5]. In outdoor air, the United States Environmental Protection Agency (EPA) specifies four principal pollutants and their corresponding maximum exposure concentrations (hourly) [6]: 9 ppm of CO, 100 ppb of NO_2_, 0.07 ppm of ozone, and 75 ppb of sulphur dioxide. If gas sensing technology is implemented into smartphones, users could assess the air quality wherever they are, perhaps allowing them to avoid unhealthy environments or, at least, indicating to them the need for ventilation. Using the built-in localization and connectivity capabilities of smartphones and wearables, any measured data could simultaneously be geo-tagged and shared to the Internet of Things (IOT) in order to build fine-grain air pollution maps.

A gas-sensitive mobile device has also a direct application to the m-Health sector (defined as medical health practice supported by mobile devices [7]). Such a device can be used to measure the concentration of VOCs present in expired breath, which might be related to respiratory and gastrointestinal dysfunction [8,9,10,11,12]. For example, CO and nitric oxide are biomarkers for asthma [12], chronic obstructive pulmonary disease [8,9], and lung cancer [11], and high levels of ammonia in expired breath are indicative of renal failure [10]. Gas sensors are less accurate than the analytical instruments usually deployed in laboratory settings or hospitals, but may be advantageous in terms of early detection of diseases, reducing healthcare costs, and promoting healthcare equity. Gas-sensitive mobile devices can also be used in healthcare applications not related to diseases. For example, tracking acetone levels in expired breath can be used to monitor sleep quality [13] and the fat-burning process [14]. Expired-air carbon monoxide (eCO) concentrations above 5 ppm might be related to smoking habits. Providing smokers with a personal monitor for measuring such concentrations was found to be a feasible method for reducing the intake of smoke [15]. Commercial eCO monitors, such as the Smokerlyzer (Bedfont Scientific Ltd. Harrietsham, UK), are already sold as plug-ins for mobile devices. Breathalyzers used to measure the blood alcohol content from breath samples are also available as plug-ins for mobile devices [16]. The connection between gas sensors and smartphones is becoming steadily more frequent. The full integration of the gas sensor into the smartphone or wearable would drastically increase the penetration of current gas-sensing applications and the appearance of new ones.

The key specifications of a gas sensor with regard to mobile integration are the cost, size, durability, stability, and power consumption. Among the different gas-sensing technologies, metal oxide semiconductor (MOX) sensors represent the most prominent technology for integration into portable devices. They have the advantages of high sensitivity to a large variety of gases, miniaturization potential, low cost, and long-life. They represent the most commercially successful type of gas sensor, and have been used in applications ranging from environmental monitoring [17,18,19,20], energy [21], food [22,23], automotive [24,25], and safety and security [26,27,28,29,30] to biomedicine [31,32]. The main limitations of this technology are their inherent lack of selectivity, high power consumption, and temporal drift [18,33,34].

**Figure 1:**
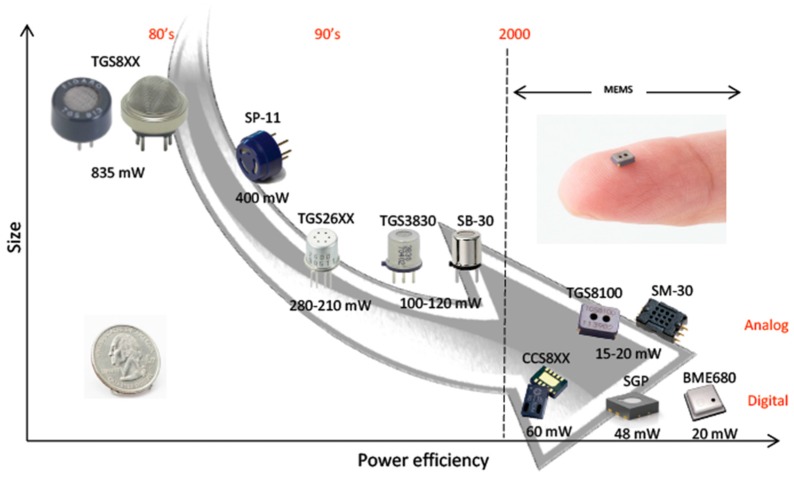
Evolution of metal oxide semiconductor (MOX) technology in terms of size and power efficiency. The background arrow indicates the temporal evolution.

The main source of power consumption in MOX sensors is the resistor that heats up the sensing surface to promote efficient redox reactions between the adsorbed molecules of the target gas and the metal oxide [35]. The evolution of fabrication technologies has permitted the miniaturization of the sensing chip and heater resistor, leading to smaller and more power-efficient sensors (see Figure 1). Thanks to micro-machined technology (MEMS), MOX sensors can be fabricated nowadays with a very compact form factor and power consumption of mere tens of mW [36].

**Figure 2:**
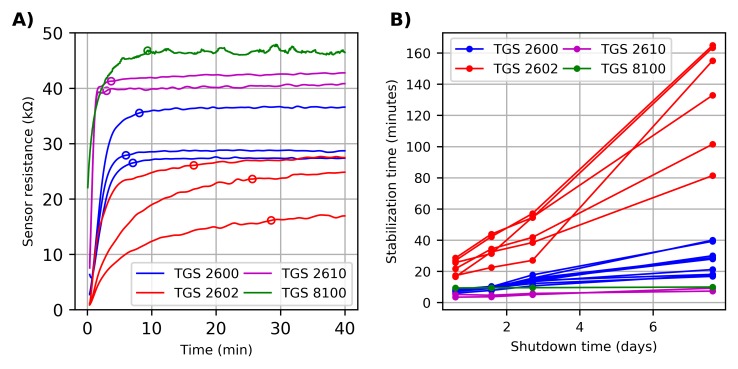
Run-in phase of several isothermally operated MOX sensors (TGS models, Figaro Engineering Inc., Arlington Heights, IL, USA). (**A**) Initial action of the sensor response after a 12-h shutdown. The open circles on top of each trace indicate the stabilization time, defined as the time when the response reaches 95% of its final value. (**B**) Stabilization time as a function of the shutdown period. The sensors were continuously powered for several days before each shutdown.

As opposed to other gas-sensing technologies (e.g., electrochemical sensors) in which power can be issued on-demand (i.e., to take a measurement), MOX sensors require a continuous energy supply. Immediately after a MOX sensor is switched on, the sensor enters into an unstable state characterized by a steady increase in the sensor resistance, known as the “run-in phase” in [37] or “initial action” in [38]. In a separate study we made, the standard duration of this transient behaviour was found to depend heavily on the sensor model and how long the sensor was unenergized (Figure 2). In general, the longer the sensor is unpowered, the longer the run-in phase takes. In some extreme cases (e.g., sensor TGS 2602), the run-in phase can take more than two hours if the sensor has been unpowered for more than one week. This means that a reliable and stable measurement can only be obtained after waiting for that length of time, which represents a practical issue for intermittently operated devices, such as portable gas detectors. Because of this, MOX sensors have traditionally been powered continuously rather than on-demand.

The power consumption under continuous power supply might be considered too high for certain battery-operated applications. As an example, consider adding a Figaro TGS8100 sensor (one of the most power efficient sensors in the market at 15 mW) to a Samsung Galaxy S7 smartphone, which consumes 462 mW under non-intensive use (see Appendix A for the details). The power consumption of the MOX sensor represents 3.14% of the total smartphone power usage or, in other words, a reduction in battery lifetime of 45 min. Considering that more than one MOX sensor might be needed to increase the selectivity of the system [39], the situation worsens. For example, the recently released SGP sensor (Sensirion AG) integrates multiple MOX sensors into the same chip, increasing the power consumption to 48 mW. This would represent 10% of the smartphone’s power consumption or a reduction in battery lifetime of 2 h and 15 min in our example. Evidently, the power consumption needs to be further reduced.

Many solutions to the problem of power consumption in MOX sensors have been proposed using the technique of duty-cycling. Duty-cycling saves energy by periodically switching on and off the sensor power and, at the same time, it achieves stability because long off periods are split into smaller “chunks”. The amount of time the sensor is powered with respect to the duration of the cycle is known as the duty cycle and this is directly proportional to the average power consumption. The duty-cycle period (or duration) can be determined by the frequency of measurements required by the application [40]. Besides this, one needs to take into account that the sensor’s stability depends on the duration of the active and inactive (or sleep) phases, which can be calculated from the duty cycle and the cycle period. The minimum duration of the active phase must be longer than the thermal time constant of the heater resistor. In other words, the heater resistor needs to be powered longer than its thermal time constant to ensure that the sensing surface will reach the optimum working temperature. Ceramic sensors have a relatively long thermal time constant, of the order of 3 s. On the other hand, the thermal response time of MEMS micro-hotplates is some 150 times faster, being of the order of 20 milliseconds [41]. The duration of the sleep phase represents a trade-off between power consumption and stability. Long inactive periods reduce the power consumption, but increase the duration of the transient behaviour that occurs after power-up (cf. Figure 2). Thus, the original duty cycle and cycle duration might need to be readjusted based on these criteria.

Previous works have explored how various combinations of duty cycle and cycle period affect the stability of the sensor response and the response time under isothermal operation [40,42,43]. Sayhan et al. [40] found that short heating pulses (below 6 s) did not work well for their proprietary ceramic sensors, but were appropriate for MEMS sensors. Recently, Macías et al. [42] proposed a 0.8% duty cycle in 10-min cycles for the detection of ethanol and cigarette smoke using ceramic Figaro TGS 26XX sensors. This is equivalent to an active phase of 5 s, which is consistent with the results found in [40]. Jelicic et al. [43] explored 20–60% duty cycles and 0.5–1.5 s periods in a MiCS-5525 sensor. They found that a duty cycle of 30% and a period of 0.5 s were optimal. In this case, the duration of the heating phase was 150 ms which is several times the thermal time constant of the sensor (20 ms). Increasing the duty cycle period from 0.5 to 1.5 s increased the limit of detection or minimum detectable concentration.

In a duty-cycling operation, the sensor response is typically measured at the end of the active phase when the response is more stable. Nonetheless, several authors have explored the performance of other features of the sensor response which might lead to lower measurement times [42,43,44,45]. Jelicic et al. [43] found an optimum measurement time of only 65 ms using features of the transient response. Rossi et al. [44] extracted features from the frequency spectrum at 20 Hz in the first 512 ms of the transient behaviour.

By definition, duty-cycling means that a given on-off pattern is repeated continuously. However, some authors have explored the response of the sensor to “bursts” of heating cycles after an inactive period [46,47]. Oletic et al. [46] explored the stability of the MiCS-5525 sensor’s resistance at the beginning (Rmin) and end (Rend) of the heating pulse after a 30 s pause. They found that Rmin was sensitive to the inactive length of the cycle, so higher duty cycles and lower periods were preferred. On the other hand, the stability of Rend depended on the energy delivered to the sensor, regardless of the duty cycle, period, or number of pulses in the “burst”. Bicelli [47] found that a burst of four pulses with high duty cycle and short period were the optimum in a Figaro TGS2442 ceramic sensor.

A common factor of the previous works is that measurements were assumed to be taken periodically. While this might be the case in certain applications, in other situations measurements are taken asynchronously. This means that a measurement can be requested at any moment even after the sensor has been switched off for a long period of time (days, weeks, or months). For example, a MOX-based breathalyser used to check the blood alcohol content in a driver’s blood might be turned on only prior to driving. Previous works also do not take possible chemical interferences into account. As Barzan et al. [48] point out, overoptimistic testing conditions in metal-oxide gas sensor research contribute to the excellent performances published. They specifically referred to the absence of changing background conditions (humidity, temperature). In gas-sensing applications in which the user can take a measurement anywhere, and at any time, it is realistic to assume that interferences such as humidity, temperature, and analytes other than the target gas will be present. Therefore, the isothermal operation of a single sensor is not reliable as MOX sensors are intrinsically not selective. Modulating the heater temperature has been shown to be an effective method for increasing selectivity [49]. Among the explored works in low-power modes for MOX sensors, only Vergara et al. [50] used temperature modulation and on-demand powering. They studied the effect of the heating and measurement time in the prediction of gases relevant to fruit quality using Figaro TGS 26XX sensors. The sensors were powered using multi-sinusoidal waveforms of length between 5 and 312 s and both the duty cycle and period were fixed. The measurements were taken periodically twice per day in a time period of 1 month. A measurement time of 39 s was found to be optimal for quantification purposes.

Following on the work of Vergara et al. [50], the current paper proposes a scenario of on-demand measurements of low concentrations of carbon monoxide with background interferences of humidity and temperature. This scenario is motivated by a myriad of applications requiring the detection of CO under variable humidity and temperature as indicated at the beginning of this section. We compare the performance of three power management strategies applied to temperature-modulated FIS SB-500-12 sensors: continuously powered (highest stability), on-demand power (lowest power consumption), and duty-cycling (a power/stability trade-off).

## 2. Materials and Methods

### 2.1. Experimental Design

**Figure 3:**
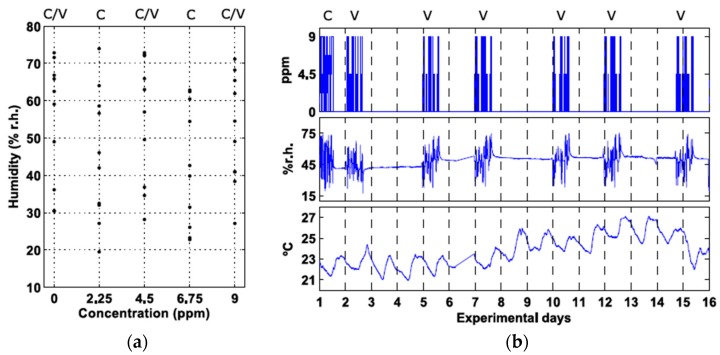
Experimental design. (**a**) Carbon monoxide and humidity values presented to the sensors in calibration and validation. “C” stands for Calibration and “V” for Validation. “C/V” means that the condition was used for both calibration and validation; (**b**) Validation plan. The subplots represent (from top to bottom) the CO concentration (ppm), the measured humidity (% relative humidity (r.h.)), and the measured temperature (°C).

The experiment timeline is shown in Figure 3. It consisted of a calibration phase, which was performed on Day 1, followed by validation measurements taken during the next two weeks. For calibration purposes, the sensors were exposed to five concentrations of CO: 0, 2.25, 4.5, 6.75, and 9 ppm. Ten measurements per day were done for each concentration, with a humidity value randomly chosen from a uniform distribution in the range 15–75% relative humidity (r.h.) (Figure 3a). We believe that this range is representative of various real scenarios, such as indoor air quality or biomedical applications based on expired breath measurements. The temperature was monitored during the two experimental weeks and it was in the range 21–27 °C. The intraday variation was below 2 °C. Dynamic gas mixtures of CO and humid synthetic air were generated using three mass flow controllers (EL-FLOW Select, Bronkhorst High-Tech B.V., Ruurlo, The Netherlands). The resulting 50 gas mixtures (5 concentrations × 10 repetitions) were randomly introduced into a small gas chamber (250 cm^3^ internal vol.) at a constant flow rate of 500 ml_n_/min for 15 min. The chamber was cleaned at the beginning of the experiment by flooding with synthetic air for 30 min at 500 ml_n_/min.

The validation experiments simulated a use case in which the measurements are requested only during the day and at random times, there being no measurements at night (the sensing device might be switched off at night) and no measurements took place on certain days (e.g., days 3 and 4). Validation measurements were performed in a similar manner to calibration, but there were two minor differences with respect to calibration: (i) only three concentration standards (0, 4.5, and 9 ppm) were used and (ii) the gas mixtures were not consecutively introduced into the gas chamber. The 30 measurements corresponding to validation (3 concentrations × 10 repetitions) were distributed within the first 16 h of the day. This yields an average elapsed time between measurements of 30 min. No measurements took place during the last 8 h of the day.

### 2.2. Sensor Board

**Figure 4:**
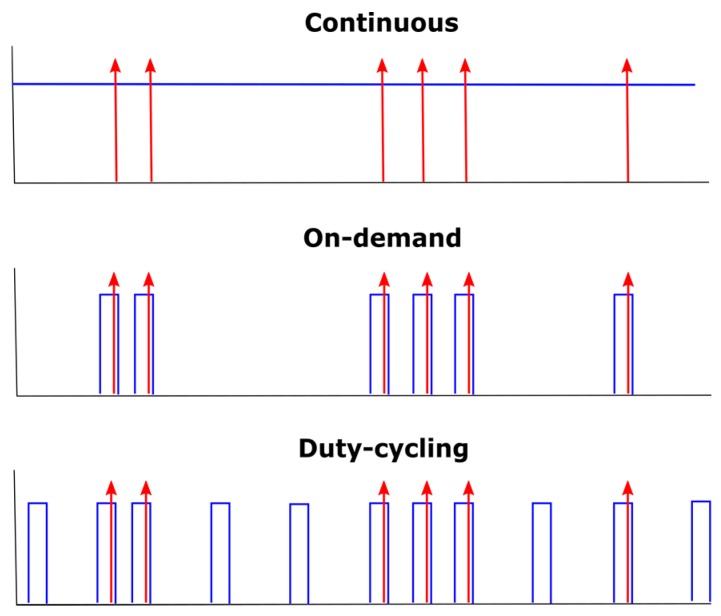
Schematic illustration of the three power management strategies used in this work. The blue solid lines represent the heater voltage and the red arrows indicate when the measurements are taken.

A board including twelve MOX sensors of the same type (SB-500-12, FIS Inc., Jacksonville, FL, USA), a temperature/humidity sensor (SHT75, Sensirion AG, Stäfa, Switzerland), and the corresponding read-out electronics was built. The FIS SB-500-12 sensors were powered using the temperature modulation waveform suggested by the manufacturer (0.9 V for 5 s, followed by 0.2 V for 20 s, in cycles of 25 s) [51]. The high heater voltage at the beginning of the cycle cleans the sensor surface and removes the water vapour influence, while the low heater voltage conditions the sensor for measuring CO. The twelve MOX sensors were divided into three groups according to the target operating mode (see Figure 4). Six sensors were operated in continuous mode (i.e., always powered), three sensors were powered on-demand (i.e., powered just before and during the measurement), and the last three sensors were operated in a duty-cycling mode with a 10% duty cycle in periods of 10 min. When any of the sensors was powered, the heating waveform suggested by the manufacturer was continuously applied. For example, a sensor operated on-demand was normally shut down and turned on only for taking a measurement. During the measurement, the heating waveform was continuously applied. For simplicity, we did not implement a closed loop control of the heater temperature although this has been shown to produce more accurate results [52]. The redundancy of sensors included in each power mode accounted for the large tolerance in baseline (one order of magnitude) and sensitivity (a factor of two) between devices [51]. By analysing the results of several units of the same model operating under a specific power mode, statistics on the performance of that mode were obtained. In duty-cycling mode, the temperature modulation waveform was applied only within the active part of the duty cycle. The duty cycle was selected so that the sensors were powered with a small number of heating cycles, as we believed this should greatly increase the stability of the sensor compared to on-demand operation. A time-ON of 60 s allowed for two full heating cycles of 25 s plus the first 10 s of a third heating cycle. Within each heating cycle, the first 5 s at 0.9 V is where the sensing surface reaches the maximum temperature and therefore is where the sensor stability increases the most. With the chosen duty cycle, the sensor was exposed to this high temperature step three times per cycle. After fixing the duty cycle at 60 s, a cycle period of 10 min was selected to obtain a reduction in power consumption of one order of magnitude. It was beyond the scope of this work to study the effect of other duty cycles. The MOX read-out circuits consisted of voltage dividers with 1 MΩ load resistors. The value of the load resistor allowed for a proper quantification of the sensor resistance, considering the large dynamic range (20 kΩ to 10 MΩ) present at low concentrations of the analyte with the chosen temperature modulation waveform. The output voltage of the sensors was sampled at approximately 3.5 Hz using an Agilent HP34970A/34901A DAQ configured at 15 bits of precision and an input impedance greater than 10 GΩ. In this configuration, the errors introduced into the voltage measurements were considered negligible compared to the intrinsic variability of the sensor resistance due to the chemical transduction process. The sensor voltage sampled at 3.5 Hz during a full heating cycle of 25 s is a multivariate signal $V_{s}\left(t\right),\;t\in \left[0,\;25\right]\;s$, containing 88 variables evenly spaced across the 25 s signal. $V_{s}\left(t\right)$ was then converted to sensor conductance $g_{s}$ (${k\Omega }^{-1}$) using Equation (1):

(1) $$g_{s}\left(t\right)=\frac{V_{c}-V_{s}\left(t\right)}{V_{s}\left(t\right)R_{L}}$$

where $V_{c}$ is the voltage (*V*) of the voltage divider and $R_{L}$ is the load resistor (kΩ). The multivariate sensor conductance patterns $g_{s}\left(t\right)$ corresponding to three consecutive heating cycles were averaged to reduce instrumental noise. This is what we call “a measurement”. When a measurement was requested, the discontinuously operated sensors were warmed up for 75 s (i.e., three full heating cycles).

The Sensirion sensor provided reference humidity and temperature values with tolerance below 1.8 % r.h. and 0.5 °C, respectively, every five seconds. According to the manufacturer datasheet [53], the long-term drift of the SHT75 sensor is less than 0.5 % r.h./year and 0.04 °C/year. The readings from the humidity sensor were averaged during the last heating period of each experimental condition to provide a reference relative humidity value *h* (% r.h.).

### 2.3. Calibration Models

Multivariate analysis was done in Matlab R2009b (The Mathworks, Inc., Natick, MA, USA) using PLS Toolbox 8.0 (Eigenvector Research Inc., Wenatchee, WA, USA). Partial Least Squares (PLS) [54] calibration models were built individually for each sensor using the measurements collected on the first experimental day. The sensor conductance patterns G were normalized by the sensor conductance in air $G_{0}$, which was estimated from a blank sample with random humidity measured at the beginning of each experimental day. The design matrix $X=G/G_{0}$ was mean-centered and auto-scaled to unit variance and the vector of responses Y was mean-centered prior to PLS modelling. The number of latent variables (LV) was optimized via 100 bootstrapping iterations on the calibration samples (see Section 2.1). In each bootstrap iteration, a PLS model was built using 50 samples randomly selected with replacement from the calibration set. The performance of the model in the set of test samples excluded from the selection process was assessed through the root mean squared error (RMSE):

(2) $$\mathrm{RMSE}=\sqrt{\overset{n}{\underset{i=1}{\sum }}\frac{{\left(y_{i}-{\hat{y}}_{i}\right)}^{2}}{n}}$$

where n denotes the size of the test set, and $y_{i}$ and ${\hat{y}}_{i}$ are the true and predicted values for the test sample i. The optimum number of LV was found by inspection of the graph of mean RMSE (across the bootstrap iterations) versus the number of LV.

The stability of the calibration models was evaluated through the root mean squared error in prediction (RMSEP) using external validation samples collected in the two weeks following calibration. The RMSEP was computed using Equation (2), but in this case n denotes the size of the external validation set and $y_{i}$ and ${\hat{y}}_{i}$ are the true and predicted values for the external validation sample i.

PLS models usually require several latent variables to account for the effect that chemical interferences produce in the sensor response patterns. When the model complexity increases beyond the chemical rank of the problem, PLS models are not easily interpretable [55]. A simpler model is advantageous to understand the underlying structure. To address this problem, the PLS models were post-processed with an orthogonalization step [56] that reduced the effective number of latent variables to two [57]. This produces simpler models with orthogonalized loadings and scores, which condense all the variance related to the target analyte into the first weight and loading. The first loading is in the same direction as the regression vector for data in which the structured noise, defined as the systematic variation of **X** not linearly correlated with **Y**, has been filtered out. The second loading of the orthogonalized model captures the variation orthogonal to Y, which is mainly related to the interferences and, to a lesser degree, other noise sources. The orthogonalized model yields the same predictions as the non-orthogonalized model.

## 3. Results and Discussion

### 3.1. Drift in Sensor Conductance Patterns

**Figure 5:**
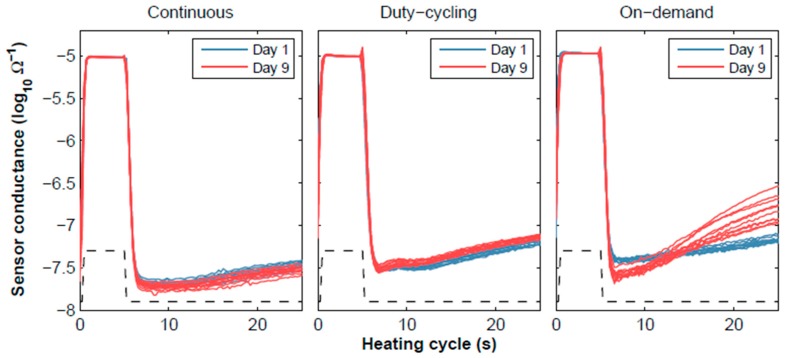
Drift in raw patterns for the three operating modes at 9 ppm of CO. The blue and red solid lines represent the sensor conductance measured during a full heating cycle taken on day 1 and day 9, respectively. The black dashed line represents the heater voltage (a.u.).

The response of the sensors to the experimental conditions described in Figure 3 was recorded for two weeks. Depending on the operating mode of each group of sensors, different trends in the sensor conductance patterns were observed (Figure 5). The patterns of the continuously operated sensors showed no relevant variations between the calibration and validation samples. The duty-cycling operation introduced a slight offset towards higher concentrations (the sensor conductance increases with concentration). The strongest deviations from the calibration data were observed in the sensors operating on-demand. In this latter case, the shape of the pattern drastically changed after nine days of on-demand operation. Although the underlying mechanisms governing this drift are still not fully understood [58], it is thought that keeping the sensing surface hot promotes water desorption and cleans any existing organic deposits from the surface [59].

### 3.2. Prediction Error

**Figure 6:**
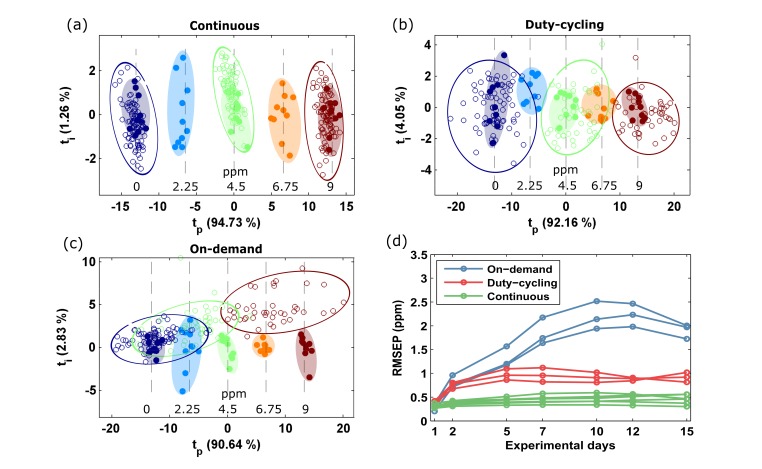
Score plot of the orthogonal Partial Least Squares (PLS) models obtained from one sensor of each group under continuous calibration. The title of the subplot indicates the target operating mode: (**a**) continuous, (**b**) duty-cycling, and (**c**) on-demand. The filled and open circles represent the calibration and validation observations, respectively. Hand drawn ellipses cluster observations of the same concentration level. The concentration (ppm) is indicated in the text at the bottom of the figure. (**d**) Temporal stability of each operating mode.

PLS models were built separately for each sensor using the conductance patterns measured on Day 1. The optimum number of latent variables was from three to five, depending on the sensor. An example of the scoreplot of the orthogonalized model of one sensor from each operating mode is given in Figure 6a–c. In these plots, the *x*-axis represents the predictive component of the model and the *y*-axis captures the variance associated to the structured noise present in the sensor conductance patterns. Regardless of the operating mode, the five calibration concentrations were non-overlapping in the score space, which indicates a good fit of the model to the data. One should note that all of the sensors were continuously powered before and during the calibration process, thus increasing their stability. The RMSEC (considering all sensors) was 0.35 ±0.05 ppm.

Immediately after calibration, the validation phase started, and the sensors were operated in the target operating mode. One would expect an increase in the prediction error introduced by the discontinuous operation (duty-cycling and on-demand modes). The error would have two components: the bias and the variance. The scoreplot of the orthogonalized PLS model is an intuitive tool to visualize these two contributions to the error term. The scores of the validation samples were projected onto the latent space of the calibration samples (Figure 6a–c). Looking at the continuous sensors (Figure 6a), the validation scores remained centred around the calibration scores (i.e., small bias), but the dispersion mainly increased mainly in the vertical direction and, only slightly, in the horizontal axis. Because the vertical axis captures the structured noise which the multivariate model is able to reject, the prediction error increased slightly (Figure 6d) due to the horizontal component of the variability. When the sensors were operated in duty-cycling mode, there was still a small bias but the variability of the scores around the calibration data was shared among the *x*- and *y*-axis (Figure 6b). Because the *x*-axis captures the predictive direction of the model, the prediction error increases (Figure 6d). Thirdly, under on-demand operation the scores presented both high bias and high variance in the predictive direction (Figure 6c). This is the result of the drastic change in the shape of the sensor conductance patterns (Figure 5).

The prediction error as a function of the elapsed time since calibration is presented in Figure 6d. On the first validation day, all of the samples were used for model building. Since there were no validation samples, the first data point represents the fitting error (i.e., the RMSE computed on the fitting samples). Over the remaining days, the RMSEP was computed on the external validation samples. It is expected that a fitted model will perform worse on unseen data than on the fitting samples [60]. This produced an increase of the average prediction error from 0.35 ppm on Day 1 to 0.43 ppm on Day 2 (22%) even under continuous operation. This differential error of 0.06 ppm, known as the “shrinkage effect”, cannot be attributed to the operating mode. For continuous operation, the average error subsequently remained stable at 0.45 ppm. The average RMSEP increase that could be attributed to the discontinuous operating modes (i.e., after discounting the shrinkage effect) was 75% (0.43 versus 0.75 ppm) for duty-cycling sensors and 97% (0.43 versus 0.85 ppm) for on-demand sensors. After the second day, the error of the duty-cycling sensors was largely stable at 0.9 ppm. Comparing this error to the error obtained under continuous powering (0.45 ppm), the trade-off between stability and power consumption can be quantified: reducing the power consumption by 90% increased the average RMSEP by only a factor of 2. On the other hand, the RMSEP of on-demand sensors kept growing for up to 9 days after calibration and then stabilized at 2.2 ppm. This represents a five-fold increase with respect to continuous power, which makes the calibration model unusable for accurate gas quantification.

### 3.3. Calibration in Discontinuous Mode

The calibration in continuous mode provided low RMSEC estimates, as the sensor response was stable throughout the calibration process. However, as we saw in the preceding section, the RMSEC was overoptimistic for the sensors that were discontinuously operated and, therefore, not representative of the detection capabilities of the system beyond the calibration data. This raises the question about the potential benefit of calibrating the sensors in the target operating mode instead of under continuous powering. To answer this question, we explored the performance when the discontinuous sensors were calibrated in the target operating mode.

**Figure 7:**
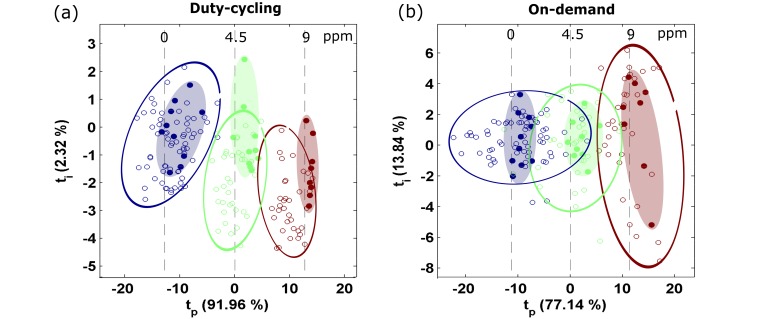
Discontinuous calibration. Score plot of the orthogonal PLS models obtained from one sensor of each group of discontinuously operated sensors. The title of the subplot indicates the target operating mode: (**a**) duty-cycling and (**b**) on-demand. The filled and open circles represent the calibration and validation observations, respectively. Hand drawn ellipses cluster observations of the same concentration level. The concentration (ppm) is indicated in the text at the top of the figure.

**Figure 8:**
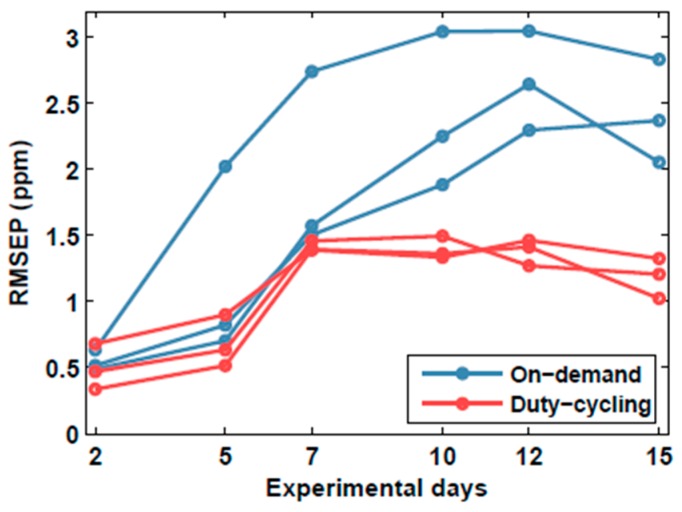
Temporal stability of duty-cycling and on-demand modes, after discontinuous calibration. RMSEP: root mean squared error in prediction.

Using the 30 samples (3 concentrations × 10 repetitions) corresponding to the second experimental day, in which the sensors were already operating in the target mode for more than 12 h, we built new PLS calibration models. The resulting RMSEC was 0.5 ±0.15 ppm, which is 43% higher than in continuous calibration. The distribution of the validation samples in the scoreplot of the calibration samples is shown in Figure 7. Comparing these scoreplots to the scoreplots obtained in continuous calibration (Figure 5b–c), we can see that in the former case the bias is greatly reduced but the variability of the blanks has increased considerably. This means that when calibration and validation are performed both under discontinuous power, the resulting response patterns are not distorted, but their variability increases. The higher bias might possibly be explained by the lower number of samples used in discontinuous calibration (30 rather than 50). With respect to continuous calibration, the average RMSEP increased 44% (0.9 to 1.3 ppm) in duty-cycling sensors and 18% (2.2 to 2.6 ppm) in on-demand sensors (Figure 8). The stabilization time was also higher in duty-cycling (2 versus 5 days) and on-demand sensors (9 versus 10 days). Therefore, we can conclude that discontinuous calibration did not improve the results obtained in continuous calibration. In addition, discontinuous calibration can be time-consuming for large duty-cycle periods. For example, assuming that 20 samples are used for calibration and the duty-cycle period is 10 min, the calibration process will take 3.3 h. In continuous mode, calibration is faster because samples can be measured consecutively.

## 4. Conclusions

MOX gas sensors are candidate devices for integration in mobile sensing devices, such as smartphones and wearables. Continuously powering a MOX sensor is not feasible in many battery-operated applications due to power consumption limitations or the intended intermittent operation of the device. This work compared two low-power operating modes (duty-cycling and on-demand) in the prediction of low concentrations of carbon monoxide under variations in ambient humidity and temperature. The sensors were modulated in temperature to increase the selectivity to the target gas and reject chemical interferences. The sensors were calibrated in continuous operation and then benchmarked for two weeks against a group of continuously powered sensors. We found that on-demand operation produced a deformation of the sensor conductance patterns, which led to an increase in the prediction error by almost a factor of 5 as compared to continuous operation (2.2 versus 0.45 ppm). Applying a 10% duty-cycling operation in periods of 10 min reduced the prediction error to a factor of 2 (0.9 versus 0.45 ppm). This means that the proposed duty-cycling powering scheme saved up to 90% energy compared to the continuous operating mode, which could be advantageous for applications that do not require continuous and periodic measurements and that can tolerate slightly higher prediction errors. We found that continuous power during calibration produces better results than calibration under discontinuous operation.

In this work, we did not test other duty cycles or periods. We expect that increasing the duty cycle will further reduce the error at the cost of higher power consumption. Future work includes exploring what is the minimum duty cycle that would achieve errors comparable to continuously operated sensors.

## Appendix A

In this appendix, we detail the computation of the power consumption in the Samsung Galaxy S7 smartphone, which hosts a 3000 mAh battery and operates at 3.7 V. The available energy (E) is therefore:

(A1) E=I × V= 3000 mAh × 3.7 V=11,100 mWh

Assuming a battery life of 24 h (non-intensive use), the average power consumption (P) is given by Equation (A2):

(A2) P=E / T= 11,000 mWh / 24 h=462 mW

Adding a TGS8100 sensor (which consumes 15 mW) to this smartphone, the total power consumption becomes 477 mW. Hence, the MOX sensor represents 3.14% of the total smartphone power. Recomputing the battery lifetime (T) using this power consumption yields:

(A3) T=E / P= 11,000 mWh / 477 mW=23.27 h

The battery lifetime is reduced by 0.73 h = 45 min. Now, we consider the case in which the SGP sensor (Sensirion AG), which consumes 48 mW, is integrated into the smartphone. The power consumption of the full system (smartphone + sensor) will be (462 + 48) = 510 mW. Introducing P = 510 mW in Equation (A3) yields that the new battery lifetime would be 21.76 h. This represents a decrease in battery lifetime of 2.23 h.
